# CBCT evaluation of buccal bone thickness in the aesthetic zone of menopausal women: A cross‐sectional study

**DOI:** 10.1002/cre2.623

**Published:** 2022-07-07

**Authors:** Nava Naghibi, Kazem Fatemi, Seyed‐Hosein Hoseini‐Zarch, Bijan Sadeghi, Mahdiye Fasihi Ramandi

**Affiliations:** ^1^ School of Dentistry, Dental Research Center, Mashhad University of Medical Science Mashhad Iran; ^2^ Oral and Maxillofacial Radiology, Dental Materials Research Center, Mashhad University of Medical Sciences Mashhad Iran; ^3^ School of Dentistry, Mashhad University of Medical Sciences Mashhad Iran; ^4^ Department of Periodontics School of Dentistry, Mashhad University of Medical Sciences Mashhad Iran

**Keywords:** buccal bone thickness, CBCT, menopause

## Abstract

**Objectives:**

Dental implants are a known treatment today. It is necessary to have at least 2 mm of bone around the implant, especially in the buccal aspect of the anterior maxilla (esthetic zone). Some systemic conditions, such as menopause, can affect the body's bone mass as well as the alveolar bone. Considering that few studies have been carried out on the effect of menopause on the thickness and topography of alveolar bone, we decided to investigate the effect of menopause on buccal alveolar bone thickness in the anterior maxillary teeth in menopausal women.

**Material and Methods:**

In this descriptive‐analytical cross‐sectional study, two subgroups of menopausal women and nonmenopausal women were considered. Data were extracted from 30 patients referred to a private radiology center in Mashhad for CBCT imaging. In addition, the buccal bone thickness in the crest and middle areas of the anterior maxillary teeth was measured and the difference between the two groups was investigated. The buccal bone thickness of the aesthetic area was evaluated with CBCT Planmeca ProMax 3D Max (Planmeca) by Planmeca Romexis 5.3.4 software, with 200 μm Voxel size and Fov 90 × 60 mm.

**Results:**

In this study, 30 women with a mean age of 49.75 ± 3.65 years in the nonmenopausal and menopausal groups were examined. It was found that the mean buccal bone thickness of the anterior maxilla in the nonmenopausal group (0.65 ± 0.25 mm) was higher than in the menopausal group (0.56 ± 0.20 mm), but the difference was not statistically significant (*p* = .2999). Only in the crestal bone of the right canine, the average bone thickness in nonmenopausal group (0.77 ± 0.33 mm) was significantly higher than the menopausal group (0.49 ± 0.22 mm) (*p* = .011).

**Conclusions:**

Owing to changes in the volume and thickness of alveolar bone in menopausal women, the thickness of the buccal bone in the aesthetic area decreases, but this is not statistically significant.

## 1 INTRODUCTION

Women experience natural menopause, which can happen at the average age of 49–51 (Zhang et al., 2016).

During menopause, the level of estrogen falls resulting in rapid bone loss and bone mass reduction (Santoro et al., 2015). Estrogen deficiency facilitates the absorption and maturation of osteoclasts and initiates the process of bone resorption due to cytokines, such as interleukin, IL‐α, interleukin6, interleukin11, and TNF‐α (Zhao, 2012) Although the effect of menopause on the skeletal system of the body has been determined, there is still controversy about the alveolar bone. Some studies report a decrease in alveolar bone volume in menopausal women (Puspitadewi et al., 2019).

In recent decades, women over the age of 50 have commonly been the candidates for implant placement, especially in the anterior and aesthetic areas.

The alveolar bone consists of two layers: (1) alveolar bone plate and (2) alveolar bone proper, also called the bundle bone; these cortical layers are separated by a trabecular bone. In a thin buccal bone plate, there is no trabecular bone in the anterior maxillary and the cortical bone is in direct contact with the alveolar bone proper. The presence of the alveolar bone proper also depends on the tooth and will disappear completely after tooth extraction. According to the studies, the bone thickness of less than 1 mm is mainly composed of alveolar bone proper and is resorbed within 4–8 weeks of tooth extraction (Pagni et al., 2012).

Studies have shown that critical buccal bone thickness should be at least 2 mm for securing favorable functional and esthetic outcomes (Huynh‐Ba et al., 2010). Some studies reported that there is a strong correlation between cortical bone thickness and implant stability (Miyamoto et al., 2005).

The most common implant complication in the thin buccal bone plates is the gingival recession (Tian et al., 2015). Alveolar bone dehiscence and fenestration lead to aesthetic problems. Also, in some cases, a lack of implant stability may lead to implant failure in the future (Kajan et al., 2020).

As a reliable system, CBCT Imaging can measure the bone thickness of the implant recipient site. It is an ideal option to examine the thickness and topography of trabecular and cortical bones separately (Fuentes et al., 2015).

Owing to the lack of studies in the field of buccal bone thickness, especially in the aesthetic area of menopausal women, the specific aim of this study was to measure the thickness of the buccal bone in the aesthetic area of menopausal women using CBCT imaging.

## 2 MATERIALS AND METHODS

This cross‐sectional descriptive‐analytical study was designed to measure the thickness of the buccal bone in the aesthetic area of menopausal women.

The study was approved by the Institutional Ethical Committee of Mashhad University of Medical Science and No. 990800 with the code IR.MUMS.DENTISTRY.REC.1399.071.

Data were extracted from 30 patients referred to a private radiology center in Mashhad, Iran. For dental therapeutic purposes, CBCT imaging was prescribed for them and buccal bone in the aesthetic zone was measured. Patients enrolled in the study were divided into two groups: nonmenopausal women (*n* = 15) and menopausal women (*n* = 15). Written informed consent was obtained. Radiographs were made with CBCT Planmeca ProMax 3D Max (planmeca) by Planmeca Romexis 5.3.4 software, with 200 μm Voxel size and Fov 90 × 60 mm. Exposure parameters were 12 s,8 mA, and 88 KVP.

Inclusion criteria included women between the ages of 45 and 55, having passed at least 1 and at most 10 years after the menopausal, presence of anterior maxillary teeth, normal periodontal, and endodontical status of anterior maxillary teeth Means There was no periapical lesion or PDL widening or any lucency. To diagnose osteoporotic cases, in addition to anterior maxillary CBCT, CBCT prescription of posterior mandible was needed.

Patients were excluded if they had a history of smoking, diabetes, osteoporosis, immunodeficiency disease, and drug assumption, such as bisphosphonates and corticosteroids, hormone therapy, or orthodontic treatment. The existence of dehiscence or fenestration in the anterior maxillary was considered an exclusion criterion.

Osteoporosis is common in menopausal patients and seems to have an effect on alveolar bone thickness (Puspitadewi et al., 2019). To eliminate the effect of the confounding agent, we excluded osteoporotic patients by the following method.

To ensure that patients were not osteoporotic, according to the study of Koh and Kim (2011) and the study of Gungor et al. (2016), indices were used as a screening tool. These indices are modified by Ledgerton's classification on panoramic images but measured in cross‐sectional CBCT radiographic images in the mental foramen area. For this purpose, horizontal parallel lines are tangential to the lower border of the mandibular cortex and the upper border of the lower cortex, the lower margin of the mental foramen, and the upper margin of the mental foramen. Indicators include CTMI^1^, CTI (I),^2^ and CTI (S).^3^ By matching these measurements with the table presented in the article, the osteoporosis status was diagnosed. Also, the CTCI index^4^ is divided into three categories: in Type A, the endosteal margin of the cortex has a regular and smooth shape; in Type B, the endosteal margin shows semicircular defects, or eventually up to two layers of endosteal debris, and Type C has abundant endosteal remnants (more than three) and is clearly porous.

By measuring the mandibular lower cortical border and matching its numbers with the Koh and Kim (2011) and Gungor et al. (2016) study tables, two osteoporotic patients were diagnosed and excluded from the study.

**Figure 1:**
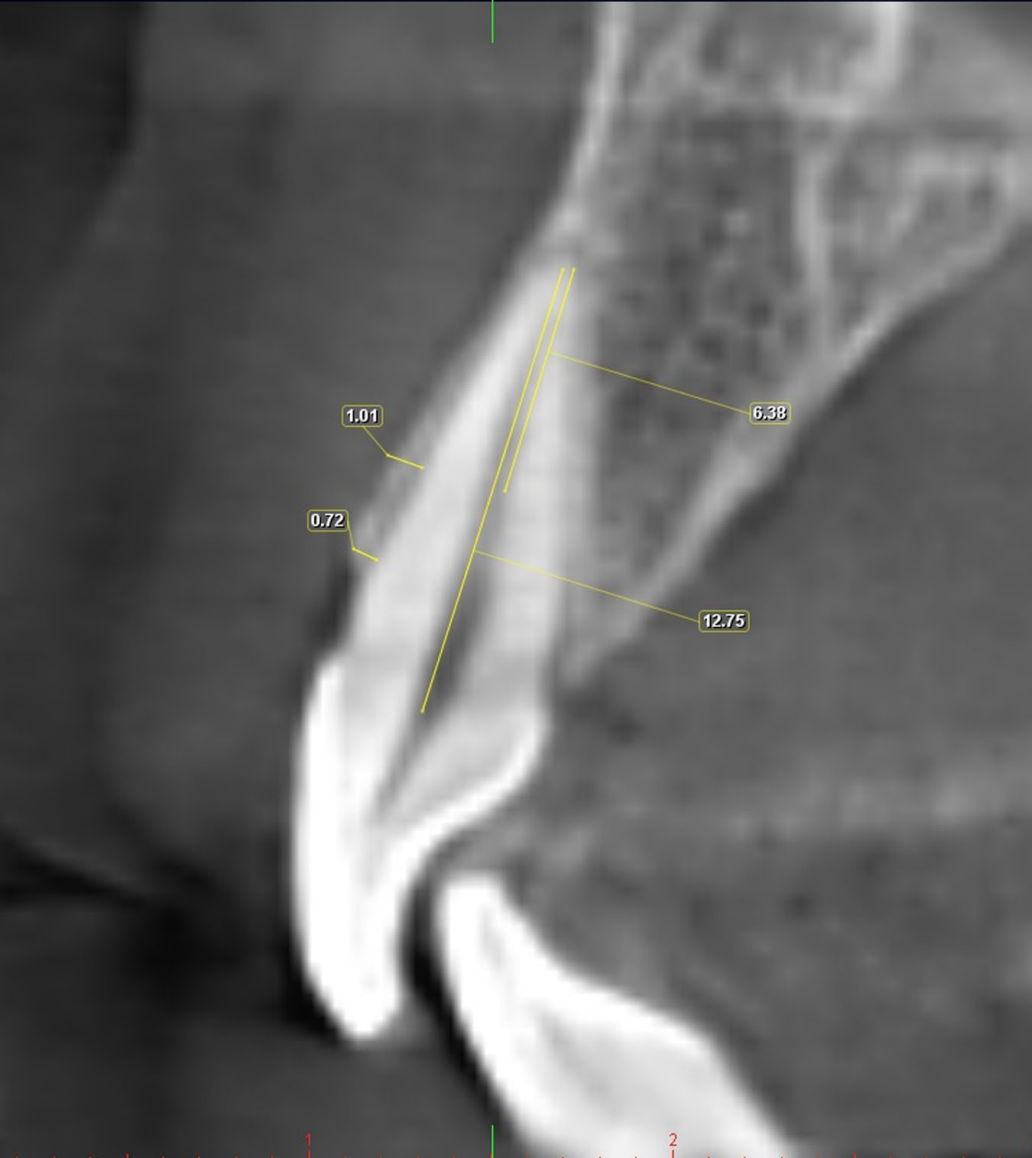
Evaluation of CBCT images from sagittal view. Maximum root length was measured from the apical point and the CEJ. Half of the measured length was determined as the midroot. Buccal bone thickness was measured at two points (crestal and midpoint).

The thickness of the buccal bone was examined by CBCT analysis by a well‐trained examiner. The CBCT image evaluation was performed as follows: the coronal and axial view was adjusted so that the maximum root length, which was measured from the apical point and the CEJ, was created in the sagittal view. Then half of the measured length was determined as the hypothetical point as the midroot. According to the study by Zhang et al. (2016), buccal bone thickness, which was the distance from the outer buccal cortex to the tooth surface, was measured by Planmeca Romexis viewer 3/3.0.R software at two points: bone crest and midroot (Figure 1).

Data analyses were performed using SPSS software (version 12). Data distribution was measured using the Shapiro‐Wilk test, which was normal. Data were reported as min–max and mean ± standard deviation (SD) bone thickness. The statistical differences among the groups were analyzed by one‐way analysis of variance. Paired *t*‐test and independent *t*‐test (or nonparametric equivalent) were used in the data analysis. *p* < .05 was considered statistically significant.

## 3 RESULTS

In this study, 30 women with a mean age of 51.93 ± 3.26 in the menopause group and 50.40 ± 2.38 in the nonmenopause group were examined for buccal bone thickness in the crest and middle areas of the anterior maxillary teeth. There was no significant difference between the groups in terms of mean age (*p* = .082).

### 3.1 Comparison of buccal bone thickness between menopausal and nonmenopausal groups in terms of area, side, and tooth

Tables 1 and 2 show the minimum, maximum, mean, and SD of bone thickness variables in the buccal area for nonmenopausal and menopausal groups and the result of the statistical test. Only in the buccal crestal bone of the upper right canine of the nonmenopausal group was significantly higher than in the menopausal group (*p* = .01).

**Table 1:** Comparison of buccal bone thickness between menopausal and nonmenopausal in terms of area, side, and tooth

Variable	Group	Number	Mean ± SD (mm)	Min–max (mm)	Mann–Whitney *U* test
Right canine buccal crestal bone thickness	Menopause	15	0.49 ± 0.22	0.10–0.90	*T *= 2.73 *p *= .011
	Nonmenopause	15	0.77 ± 0.33	0.24–0.29	
Right lateral buccal crestal bone thickness	Menopause	15	0.69 ± 0.39	0.28–1.67	*T *= 0.10 *p *= .923
	Nonmenopause	15	0.70 ± 0.27	0.24–1.013	
Right central buccal crestal bone thickness	Menopause	15	0.61 ± 0.17	0.30–0.95	*T *= 0.05 *p *= .964
	Nonmenopause	15	0.62 ± 0.29	0.21–1.05	
Left central buccal crestal bone thickness	Menopause	15	0.56 ± 0.20	0.25–0.92	*T *= 1.28 *p *= .212
	Nonmenopause	15	0.65 ± 0.18	0.31–1.09	
Left lateral buccal crestal bone thickness	Menopause	15	0.56 ± 0.24	0.20–0.96	*Z *= 0.87 *p *= .383
	Nonmenopause	15	0.71 ± 0.40	0.30–2.04	
Left canine buccal crestal bone thickness	Menopause	15	0.63 ± 0.39	0.21–1.25	*Z *= 0.04 *p* = .967
	Nonmenopause	15	0.56 ± 0.25	0.30–1.15	
Right canine buccal midroot bone thickness	Menopause	15	0.39 ± 0.22	0.11–0.90	*Z *= 1.59 *p *= .110
	Nonmenopause	15	0.64 ± 0.45	0.20–0.74	
Right lateral buccal midroot bone thickness	Menopause	15	0.59 ± 0.44	0.15–1.75	*Z *= 0.08 *p *= .934
	Nonmenopause	15	0.55 ± 0.35	0.20–1.29	
Right central buccal midroot bone thickness	Menopause	15	0.60 ± 0.30	0.32–1.24	*Z *= 0.21 *p *= .835
	Nonmenopause	15	0.58 ± 0.25	0.20–1.10	
Left central buccal midroot bone thickness	Menopause	15	0.49 ± 0.25	0.16–1.09	*T *= 1.73 *p *= .094
	Nonmenopause	15	0.66 ± 0.26	0.21–1.09	
Left lateral buccal midroot bone thickness	Menopause	15	0.51 ± 0.31	0.15–1.14	*Z *= 0.50 *p *= .619
	Nonmenopause	15	0.59 ± 0.44	0.15–1.81	
Left canine buccal midroot bone thickness	Menopause	15	0.57 ± 0.38	0.15–1.53	*Z *= 1.06 *p *= .290
	Nonmenopause	15	0.65 ± 0.28	0.30–1.08	

**Table 2:** Bone thickness in the buccal area for nonmenopausal and menopausal groups

Side	Area	Group	Thickness (min–max)	Mean ± SD	Mann–Whitney *U* test
Right	Crest	Menopause	0.29–1.04	0.60 ± 0.21	*T *= 1.13 *p *= .269
		Nonmenopause	0.28–1.13	0.70 ± 0.26	
	Midroot	Menopause	0.26–1.05	0.53 ± 0.23	*Z *= 0.58 *p *= .561
		Nonmenopause	0.21–1.15	0.59 ± 0.30	
Left	Crest	Menopause	0.33–1.04	0.59 ± 0.22	*Z *= 0.77 *p *= .443
		Nonmenopause	0.42–1.20	0.64 ± 0.23	
	Midroot	Menopause	0.23–1.11	0.53 ± 0.25	*Z *= 1.43 *p *= .153
		Nonmenopause	0.32–1.28	0.56 ± 0.28	

### 3.2 Comparison of buccal bone thickness between groups by area

Table 3 shows the thickness of the bone in the buccal crestal and midroot of the anterior teeth in the nonmenopausal and menopausal groups. The mean thickness of buccal bone in both crest and middle areas of the anterior teeth in the nonmenopausal group was higher than in the menopausal group, but the difference was not statistically significant.

**Table 3:** The thickness of the bone in the buccal crestal and midroot of the anterior teeth in the nonmenopausal and menopausal groups

Area	Group	Thickness (min–max)	Mean ± SD	Mann–Whitney *U* test
Crest	Menopause	0.37–0.99	0.59 ± 0.20	*T *= 0.98 *p* = .333
	Nonmenopause	0.38–1.14	0.67 ± 0.23	
Midroot	Menopause	0.25–0.99	0.52 ± 0.24	*Z *= 0.97 *p* = .329
	Nonmenopause	0.34–1.16	0.62 ± 0.27	

### 3.3 Comparison of mean buccal bone thickness between groups

Table 4 shows the buccal bone thickness in the nonmenopausal and menopausal groups. The mean buccal bone thickness in the nonmenopausal group was higher than in the menopausal group, but the difference was not statistically significant (*p* = .299).

**Table 4:** Buccal bone thickness in the nonmenopausal and menopausal groups

Group	Thickness (min–max)	Mean ± SD	Mann–Whitney *U* test
Menopause	0.36–0.94	0.56 ± 0.20	*Z *= 1.04 *p* = .299
Nonmenopause	0.36–1.15	0.65 ± 0.25	

## 4 DISCUSSION

In this cross‐sectional study, 30 patients with an average age of 49.77 years were examined. According to the results of our study, in the maxillary anterior teeth, the buccal bone thickness in the crest and middle of the root in menopausal women was less than in nonmenopausal women, which was not statistically significant. However, the only statistically significant difference was observed in the crest of the right canine, which was 0.49 and 0.77 in menopausal women and nonmenopause, respectively. This difference can be explained by the cross‐sectional and retrospective nature of the study where incidental increase could be observed in canine bone thickness.

In the present study, there was no significant difference between the right and left maxillary bone thickness teeth in the two groups of menopausal and nonmenopausal women. Jin and coworkers also examined the thickness of the maxillary canine crestal bone. They did not report any significant differences between the right and left as well as between men and women at the measured points (Jin et al., 2012).

In 2020, Tsigarida and coworkers performed a meta‐analysis to examine the buccal bone thickness in the anterior maxilla. In this meta‐analysis, the average bone thickness in the middle of the root and crestal bone of anterior maxillary in women were 1.6–1.7 and 0.05–1.3 mm respectively, which were larger than the average thickness of bone in the middle of the root (0.52–0.62 mm) and crestal bone (0.59–0.67 mm) of the present study (Tsigarida et al., 2020).

Zhang and coworkers performed a study on 239 patients, including 59 menopausal women, 60 nonmenopausal women, and 60 men. They stated that the bone thickness in the crest and middle buccal area of the anterior maxillary in menopausal women was significantly less than the nonmenopausal women. Also, in the study, buccal bone thickness was reported more than 1 mm in most areas which is contrary to the present study with the averages of buccal bone thickness measured 0.56–0.65 mm (Zhang et al., 2016).

The results of several studies indicate that aging leads to changes in bone metabolism and consequently a decrease in bone mass. In the present study, by comparing the mean age of the two groups of menopausal and nonmenopausal women, the effect of age as the confounding factor was removed. Therefore, the reason for the difference in the results of the present study and Zhang and coworkers' study can be attributed to the difference in the mean age of the nonmenopausal women in the two studies. In the present study, the mean age of the menopausal women was 51 years and for nonmenopausal women, it was 50 years. Accordingly, the mean age of the menopausal group was 62 years and the nonmenopausal age was 28 years. On the other hand, the geographical location and race of the studied women can alter the results (Zhang et al., 2016).

Ko and coworkers studied the association between menopausal age and crestal bone thickness of the implant site. They examined 85 patients including 48 women under 50 years and 37 women over 50 years. In their retrospective study, the authors could not accurately determine the status of women's menopause so they used age classification and considered women over 50 years old as menopausal and women under 50 years old as nonmenopausal. Although the bone thickness was lower in women over 50 years than in women under 50, they could not find a significant difference between the bone thickness and women's age, which was in agreement with the present study (Ko et al., 2020).

Mikami and coworkers performed a study with the aim of comparing the volume and microstructure of maxillary and mandibular bone mass on 18 Japanese women, including 5 nonmenopausal women with a mean age of 37.6 years, 3 women in primary stages of menopause with an average age of 54.3 years, and 10 women who passed more than 10 years from the menopause onset, with an average age of 65. They also reported that after the menopausal onset, alveolar bone structural changes. They stated that bone resorption in menopausal women is more severe and faster. Contrary to the present study, Mikami reported that the volume of bone mass in menopausal women is significantly less than in nonmenopausal women. The differences in results can be attributed to the variation in the methodology of bone mass and bone volume assessment, where we measured the buccal bone thickness with CBCT while Mikami and coworkers used morphometric methods and bone metabolism markers (Yamashita‐Mikami et al., 2013).

From the findings of this study, it can be concluded although it was not statistically significant, the average alveolar bone thickness in the buccal area in the menopause group was slightly lower than in the nonmenopause group.

Future studies can be conducted with a larger sample size and in a multicenter study on different populations in terms of race. They can simultaneously examine the effect of the number of years after menopause and measure biotype and soft tissue thickness on bone thickness.

Patients with osteoporosis should be diagnosed by examining laboratory tests and densitometry parameters. Also, to increase accuracy, along with radiographic examinations, histological examinations should be performed.

## 5 CONCLUSION

It was found that although buccal thickness was not statistically significant between the two groups, the mean buccal bone thickness in the anterior maxillary was less in the menopause group. Therefore, if necessary, in anterior maxillary edentulousness, appropriate treatment methods should be considered for each patient.

## AUTHOR CONTRIBUTIONS

**Nava Naghibi**: conceptualization, methodology, review & editing. **Kazem Fatemi**: conceptualization, methodology, review & editing. **Seyed‐Hosein Hoseini‐Zarch**: methodology, resources. **Bijan Sadeghi**: investigation, original draft, formal analysis. **Mahdiye Fasihi Ramandi**: investigation, writing—original draft.

## CONFLICT OF INTEREST

The authors declare no conflict of interest.

## Data Availability

The data that support the findings of this study are available from the corresponding author upon reasonable request.

## Footnotes

1 Lower cortical width of the mandible.

2 Ratio of CTMI to the distance between the lower margin of the foramen mental and the lower border of the mandible.

3 Ratio of CTMI to the distance between the upper margin of the foramen mental and the lower border of the mandible.

4 Type of mandibular lower cortex.
